# Policy analysis of system responses to addressing and reversing the obesity trend in China: a documentary research

**DOI:** 10.1186/s12889-023-15890-7

**Published:** 2023-06-21

**Authors:** Yan Xue, Zhen Ruan, Carolina Oi Lam Ung, Yunfeng Lai, Hao Hu

**Affiliations:** 1 State Key Laboratory of Quality Research in Chinese Medicine, Institute of Chinese Medical Sciences, University of Macau, Macao SAR, China; 2 Department of Public Health and Medicinal Administration, Faculty of Health Sciences, University of Macau, Macao SAR, China; 3 School of Public Health and Management, Guangzhou University of Chinese Medicine, Guangzhou, China

**Keywords:** Obesity, China, Health policy, Health System

## Abstract

**Background:**

The obesity epidemic and its established consequences on population health and the economy call for a vigorous fight against excess weight. The primary aim of this study is to investigate China’s responses to address and reverse the obesity trend by analysing the official documents and guidelines issued or coordinated by the central government through the lens of the WHO Health System Six Building Blocks.

**Methods:**

This study is based on the method of document research. We screened the official documents obtained through the initial search on databases. The basic information of the included documents was tabulated, and the relevant content was mapped onto the analytical framework for further analysis.

**Results:**

The screening process finally rendered 55 out of 152 official documents. The temporal distribution of these documents issued between 2003 and 2022 concentrated in the period starting 2016 until now. The State Council and its affiliated ministries were found to play an instrumental role in the efforts to tackle the obesity epidemic. The results from further analysis based on the framework revealed that the current government responses to the obesity epidemic were mainly oriented towards strengthening governance, promoting healthcare delivery to prevent obesity, and improving access to interventions. The components of financing, information system, and workforce are currently absent in the relevant documents.

**Conclusions:**

Our analysis indicated that China’s health system has taken action in response to the unprecedented obesity prevalence in recent years. In preventing and managing obesity and overweight in the population, the government affirmed its central place in governing and coordinating different recourses. The promotion of healthcare service delivery and access to medical products and technologies have been reflected gradually in the relevant policy documents. An integrated endeavour should be made in the future from all six aspects of the health system to halt the further rise in overweight and obesity.

**Supplementary Information:**

The online version contains supplementary material available at 10.1186/s12889-023-15890-7.

## Background

Obesity has been highlighted as a public health issue since the 1980s, which first emerged in high-income countries and more recently challenged the globe [1–4]. The projection of obesity or overweight prevalence in a predictive modelling study was about 50% of the world population in 2030 [5]. Scientific evidence has been mounting to testify that obesity is a complex, progressive, and frequently relapsing disease, as a result of a complicated combination of genetic, lifestyle, socio-economic and environmental factors, which may bring tremendous harm to health [1, 6–8]. The rise of its prevalence at an alarming rate and its intrinsic linkages with many other chronic health conditions have shaped obesity as one of the leading causes of aggravating the burdens of healthcare systems worldwide [9, 10].

In China, the rapid contemporary urbanisation process in the past decades has fueled a more obesogenic environment [11, 12]. The overall upward trend of obesity or overweight was seen in different groups of the population, which was supported by studies of data in various periods in the recent 30 years [13–17]. A recent meta-analysis and projection analysis based on cohort studies revealed that the combined overweight and obesity prevalence would reach 70.5% respectively in the adult in 2030 [18]. In another projection analysis based on large-scale survey results, the prevalence of overweight and obesity among school-aged children (7–17 years old) demonstrated a persistent up and was estimated to reach 31.8%, while that in the pre-school group showed a fluctuation but overall rise to 12.8% by 2030 [19]. Alongside, a series of relatively large-scale studies on the disease burden in the Chinese have emerged in recent years to testify the close association of obesity with many other chronic or critical health conditions [18, 20–25]. Subsequently, the substantial financial burden of this complex disorder on the healthcare system is unfolding. People with overweight or obese groups incurred significantly higher total direct health care costs compared with the normal-weight population based on the China Health and Retirement Longitudinal Study (CHARLS) in 2011 [26]. Early in 2003 with the combined prevalence of overweight and obesity at around 30% of the population, the medical costs attributable to overweight and obesity were estimated to represent 3.7% of national total medical costs in that year [27]. While by 2030, the percentage was projected to reach 22% of the total national medical cost [28].

This ongoing obesity epidemic has prompted to take accelerated responses to the prevention and management of obesity to halt the further increase and eventually reserve the trend. Literature documented the efforts and progresses in the countries’ policies to combat obesity [29–39]. In China, there were only a few studies on the policy options and interventions initiated by the government. Prevention-related policy measures taken by the government between 2002 and 2013 were generally reviewed [40]. In a more recent study, the current state of obesity-related policies and programmes was very briefly introduced. In another article, nationwide intervention programmes to prevent childhood obesity in China spanning from 1949 to 2021 were examined systematically [41]. However, there is a lack of systematic policy analysis of obesity-related official documents from the perspective of health policy and systems research (HPSR).

Therefore, we aim to closely evaluate the existing national official documents related to the prevention and control of obesity to understand how China’s health system has worked towards the obesity crisis. Through the lens of a practical framework of the WHO Health System Six Building Blocks, we expect to obtain a comprehensive understanding of the current strengths and challenges in the health system to combat obesity in the population.

## Methods

This study is based on the method of document research. We applied the common practice of the READ approach in the review procedures by preparing the materials, extracting the content, performing the analysis, and summarising the findings [42]. We first searched and screened the official documents based on the eligibility criteria. Next, an iterative process of skimming and reading the included documents was undertaken to extract the pertinent content for further examination. Then, the basic characteristics of the included documents together with the mapping results of the relevant content against the analytical framework were tabulated for a textual summary.

### Data sources and search strategy

To obtain a relatively exhaustive list of national official documents and relevant guidelines about weight control, prevention, and management of obesity and overweight via the initial search, we utilised three main channels for us to obtain the relevant documents, namely, *Zhengyantong*, official websites of the National People’s Congress (NPC), State Council and its affiliated ministries, as well as the key references we identified. *Zhengyantong*(www.zytdata.com/) is a domestic policy database for the search and analysis of official documents at various levels. The keywords in the searches to identify the relevant documents are three simple Chinese words, including *tizhong* (weight), *feipang* (obesity), and *chaozhong* (overweight).

We first performed a search of the three keywords in both title and full text by selecting all the government departments for a comprehensive search within *Zhengyantong*. Also, we conducted a round of searches through the official websites of all the relevant government departments (see Supplementary File 1). As the built-in searching function of these websites mainly allows keywords in a limited number of characters without Boolean operators, we conducted searches of each of the three keywords in the content respectively on the websites. Additionally, we conducted hand searching of relevant documents mentioned or included in the key references for the purpose of cross-checking. All the searches were completed on 18 June 2022.

### Eligibility criteria

We consider the following criteria in the selection of eligible documents:

#### Inclusion criteria

- The official document was issued since the existence of the data sources.
- The official document was issued by the agencies at the central government level, including the NPC, State Council and its affiliated ministries or subdivisions.
- The preparation of documents (e.g., guidelines) involved the affiliated ministries or subdivisions of the State Council.
- The content containing the keywords was about obesity and overweight prevention and control.

#### Exclusion criteria

- The document content was restricted to a specific region.
- The document content containing the keywords was not relevant to obesity and overweight prevention and control.

### Data screening

After combining the search results from the three major channels on the literature management software *EndNote*, we first removed the duplicates by applying the function of de-duplication and manual operation. We then screened the content about obesity and overweight in the remaining documents. In this process, two researchers independently reviewed the policy documents for final inclusion into further analysis. Any disagreements were resolved through discussion. It mainly involved removing the documents containing the keywords though without specific content relevant to obesity control and management.

### Content extraction, tabulation of information and content mapping

We tabulated the basic information of each document together with the relevant content in a data extraction form, covering the full title of the document, year of issuing, issuing agency and document number. For our in-depth analysis, we added different columns in the excel file for further labelling by categories, which mainly include the period of the Five-Year Plan, document type, whether involving intersectoral collaboration, and policy approach. Moreover, we extracted the relevant document content and further mapped them to the framework from the perspective of the WHO Health Systems.

### Analytical framework

In consistency with the aim of the study, we adopted the WHO Health System Building Blocks to distil and conceptualise our findings. HPSR has received remarkable research attention mainly because the health system strengthening is considered as the core to achieving tremendous advancements in all the health systems at different levels and promoting population health [43, 44]. The WHO Health System consists of six building blocks: leadership/governance, service delivery, medical products and technologies, health workforce, information system, and health financing [43, 45]. The convergence of these six blocks would contribute to the improvement of the health system as a whole. The health system angle is expected to facilitate us in the understanding of how the health system revolving around obesity prevention and control functions and how it could be strengthened by making better linkage to the general health system. We utilised the framework in the iterative process of identifying pertinent content and follow-up analysis.

## Results

### Inclusion of documents

**Fig. 1:**
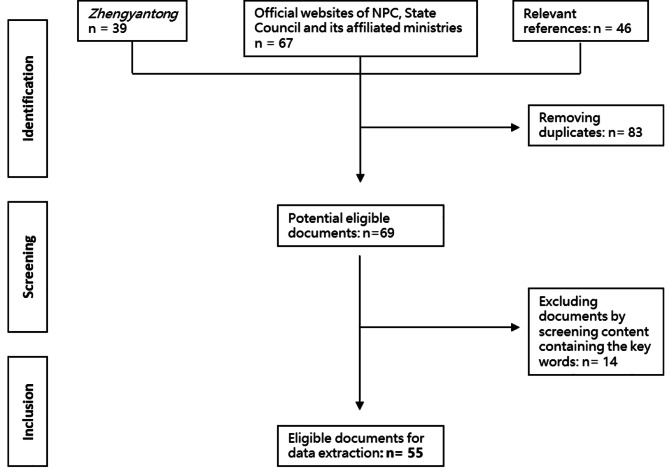
The flowchart of data identification, screening and inclusion

Our initial searches generated 152 documents (Fig. 1). After removing 83 duplicates, we further screened the remaining 69 documents based on the eligibility criteria for potential inclusion. Finally, we included 44 official documents issued by the state council and/or various ministries, together with 11 guidelines or reports issued or led by various sectors at the central level (see Supplementary File 2 for the full list of documents included with titles in Chinese and English).

### General characteristics

#### Temporal distribution and document type

**Fig. 2:**
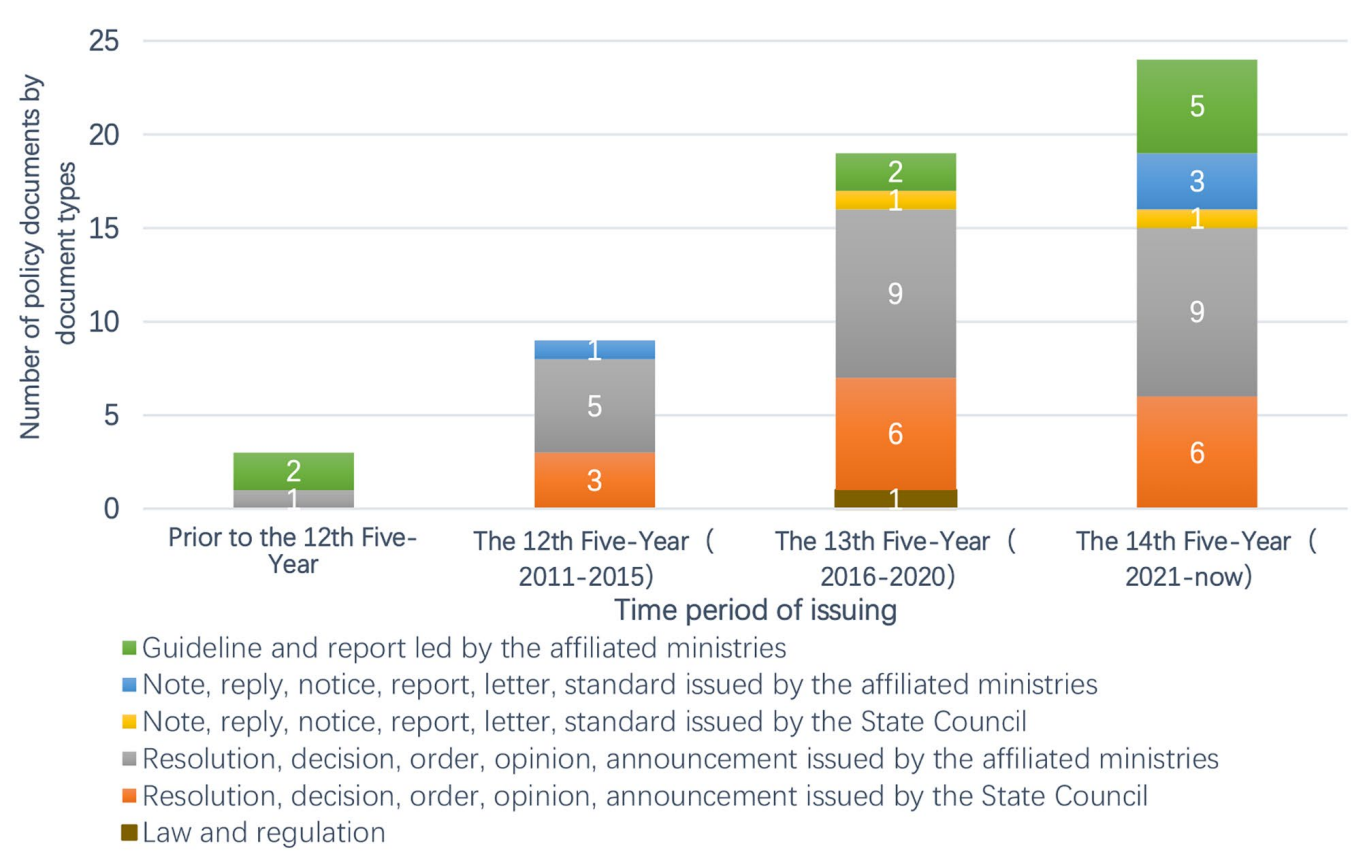
Documents categorised by document type and time of issuing

The numbers of official documents were observed to increase significantly along with time. In China, the Five-Year Plan is a key policy process, which has been formulated every five years as a consistent mechanism to guide China’s development in all aspects strategically since its first initiation in 1953. The latest one was issued in 2021, and entitled “Outline of the 14th Five-Year Plan (2021–2025) for National Economic and Social Development and the Long-Range Objectives Through the Year 2035”. Herein, we grouped the documents based on different Five-Year Plan periods (see Fig. 2). From 2003 to the beginning of the 12th Five-Year in 2011, there were only three relevant documents. Between 2011 and 2015, nine documents were launched. The next five years witnessed a doubled number of official documents at the central government level. Since the inception of the 14th Five-Year in 2021, there have been 24 official documents containing content about obesity prevention and control.

We then categorised the 55 documents into six major types (as listed in the legend of Fig. 2). The first category includes laws and regulations issued by the NPC. The second and third categories comprise resolutions, decisions, orders, opinions, and announcements issued by the State Council and its affiliated ministries. The fourth and fifth categories include notes, replies, notices, reports, letters, and standards issued by the State Council and its affiliated ministries. The last category mainly contains the guidelines and reports led by the affiliated ministries of the State Council. It could be seen that almost 3/4 of official documents fell into the second and third categories. When we combined the categories with a period, there was a clear increase pattern within each type of document since the 13th Five-year Plan Period. As the categories of documents reflect the policy intensity, the numeric pattern distributed in different periods indicated the enhanced focus on obesity and overweight prevention and control at the national level.

#### Key government agencies

According to the data in Fig. 2, the affiliated ministries of the State Council were found to play a pivotal role in the efforts of tackling the obesity epidemic, as they are the pillars of policy planning and implementation within the central government. Among all the affiliated ministries of the State Council, the National Health Commission (including the then Ministry of Health, National Health and Family Planning Commission) occupies the leading position in most of the official documents covering content of obesity and overweight prevention and control. It released 23 obesity-related official documents independently or jointly with other ministries or affiliated departments. It also coordinated the production of five documents with professional research agencies.

Among the documents issued by or involving the affiliated ministries, most of the documents were released by a single agency (see Table 1). As the prevention and control of obesity is supposed to require systematic efforts, inter-departmental collaboration is a necessary and effective approach, reflected in the eight official documents engaging several different ministries and their subdivisions.

**Table 1:** Number of Documents by Category: Whether Involved Single or Multiple Issuing Agencies

Category of Documents	Issuing Agencies	
	Single	Multiple
• **Resolution, decision, order, opinion, announcement issued by the affiliated ministries**	17	7
• **Note, reply, notice, report, letter, standard issued by the affiliated ministries**	3	1
• **Guideline and report led by the affiliated ministries**	5	0
**Total Number**	25	8

### Policy mapping and analysis of the included documents against the WHO Health System Building Blocks

To obtain a clear overview of the composition of the health system in the combat of obesity and overweight, we mapped the content extracted from the included documents onto the WHO Health System Building Blocks (see the details in Table 2).

**Table 2:** Policy Mapping of Document Content Against the WHO Health System Building Blocks

Document No	Document Title in English	Year of Issue	WHO Health System Building Blocks					
			Service Delivery	Information System	Health Workforce	Medical Products & Technologies	Health Financing	Leadership/ Governance
**1**	Guidelines for Prevention and Control of Overweight and Obesity in the Chinese Adults (2003)	2003	1	0	0	1	0	0
**2**	Guidelines for Prevention and Control of Overweight and Obesity in Chinese School-age Children and Adolescents (Trial)	2007	1	0	0	1	0	0
**3**	Management Measures for Nutrition Improvement	2010	1	0	0	0	0	1
**4**	Outline of Chinese Child Development (2011–2020)	2011	0	0	0	0	0	0
**5**	National Fitness Program (2011–2015)	2011	0	0	0	0	0	0
**6**	China’s Chronic Disease Prevention and Control Work Plan (2012–2015)	2012	1	0	0	0	0	1
**7**	Technical Specifications for the Management of Nutritional Diseases in Children” (including “Technical Specifications for the Guidance of Child Feeding and Nutrition”	2012	0	0	0	0	0	0
**8**	Maternal and Child Health Literacy-Basic Knowledge and Skills (Trial)	2012	0	0	0	0	0	0
**9**	Outline of Food and Nutrition Development in China (2014–2020)	2014	0	0	0	0	0	0
**10**	National Student Physical Health Standard (Revised in 201	2014	0	0	0	0	0	0
**11**	Chinese Citizens’ Health Literacy, Basic knowledge and Skills Definitions (2015)	2015	0	0	0	0	0	0
**12**	Report on Chinese Residents’ Chronic Diseases and Nutrition 2015	2015	0	0	0	0	0	0
**13**	Outline of Healthy China 2030” Plan	2016	1	0	0	0	0	1
**14**	“Thirteenth Five-Year” Medicine and Health Plan	2016	1	0	0	0	0	1
**15**	Medium- and Long-Term Plan for the Youth Development (2016–2025)	2016	0	0	0	0	0	0
**16**	Guidance on strengthening health promotion and education	2016	0	0	0	0	0	0
**17**	Dietary Guidelines for Chinese Residents (2016)	2016	0	0	0	0	0	0
**18**	Dietary Guidelines for Pregnant Women (2016)	2016	0	0	0	0	0	0
**19**	China’s Medium- and Long-Term Plan for the Prevention and Treatment of Chronic Diseases (2017–2025)	2017	1	0	0	0	0	1
**20**	National Nutrition Plan (2017–2030)	2017	1	0	0	0	0	0
**21**	Health Action Plan for All (2017–2025)	2017	0	0	0	0	0	0
**22**	Guidelines for National Physical Fitness	2017	0	0	0	0	0	0
**23**	Action Plan for Healthy Children (2018–2020)	2018	1	0	0	0	0	0
**24**	Core Information and Interpretation of Chinese Youth Health Education	2018	0	0	0	0	0	0
**25**	Screening for Overweight and Obesity in School-age Children (WS / T 586–2018)	2018	1	0	0	0	0	0
**26**	Guidelines for Consumption of Snacks for Chinese Children and Adolescents	2018	0	0	0	0	0	0
**27**	School Food Safety and Nutrition and Health Management Regulations	2019	0	0	0	0	0	1
**28**	Healthy China Initiative (2019–2030)	2019	1	0	0	0	0	0
**29**	Opinions of the State Council on Implementing Healthy China Action	2019	0	0	0	0	0	1
**30**	Basic Healthcare and Health Promotion Law	2019	0	0	0	0	0	1
**31**	Notice of State Council General Office on Printing and Distributing the Outline for the Construction of a Powerful Country in Sports	2019	0	0	0	0	0	0
**32**	Implementation plan for prevention and control of obesity in children and adolescents (2020)	2020	1	0	0	0	0	1
**33**	Opinions on Stepping up Patriotic Health Campaigns and Efforts	2020	1	0	0	0	0	0
**34**	Report on Chinese Residents’ Chronic Diseases and Nutrition 2020	2020	0	0	0	0	0	0
**35**	Major Work Plans for Deepening the Reforms of the Medical and Health System of 2021	2021	1	0	0	0	0	0
**36**	“Fourteenth Five-Year” Plan for Establishing the National Capacity of Clinical Specialties	2021	1	0	0	0	0	0
**37**	Special actions such as “three reductions and three health benefits”	2021	1	0	0	0	0	0
**38**	Implementation Plan for Consolidating and Building on the Effective Connection Between the Achievements of Poverty Alleviating Efforts Through Healthcare and Rural Revitalisation	2021	0	0	0	0	0	1
**39**	Notice of the NHC on Holding Series of Awareness Days of Non-Communicable Diseases	2021	1	0	0	0	0	0
**40**	Enhanced Action Plan for Healthy Children (2021–2025)	2021	1	0	0	1	0	0
**41**	Outline of Chinese Children Development (2021–2030)	2021	1	0	0	0	0	0
**42**	Outline of Chinese Women Development (2021–2030)	2021	1	0	0	0	0	0
**43**	National Fitness Program (2016 ~ 2020)	2021	0	0	0	0	0	0
**44**	Technical Guiding Principles for the Clinical Trials of Body Weight Control Drugs	2021	0	0	0	1	0	0
**45**	Management Measures of Health Examination in Primary and Secondary Schools (2021)	2021	0	0	0	0	0	0
**46**	China Blue Paper on Obesity Prevention and Control	2021	1	0	0	1	0	0
**47**	Expert Consensus on the Procedure of Body Weight Management Among Patients with Overweight or Obesity	2021	1	0	0	1	0	0
**48**	Guidelines for Prevention and Control of Overweight and Obesity in Chinese Children (Trial)	2021	1	0	0	1	0	0
**49**	Guidelines for Prevention and Control of Overweight and Obesity in the Chinese Adults (2021)	2021	1	0	0	1	0	0
**50**	Notice of State Council General Office on Printing and Distributing the “Fourteen Five-Year” Plan for the Traditional Chinese Medicine Development	2022	1	0	0	1	0	0
**51**	“Fourteenth Five-Year” National Health Plan	2022	1	0	0	0	0	0
**52**	“Fourteenth Five-Year” Plan for Promoting the Health Standardisation Work	2022	1	0	0	0	0	0
**53**	Guiding Principles for the Design of Clinical Trials of Liraglutide for Weight Management	2022	0	0	0	1	0	0
**54**	Dietary Guidelines for Chinese Residents (2022)	2022	0	0	0	0	0	0
**55**	Implementation Plan for the Special Campaign on Promoting Healthy China	2022	0	0	0	1	0	0

**Note: 1 = relevant content found in the extracted content from the included document; 0 = no relevant content**

**Fig. 3:**
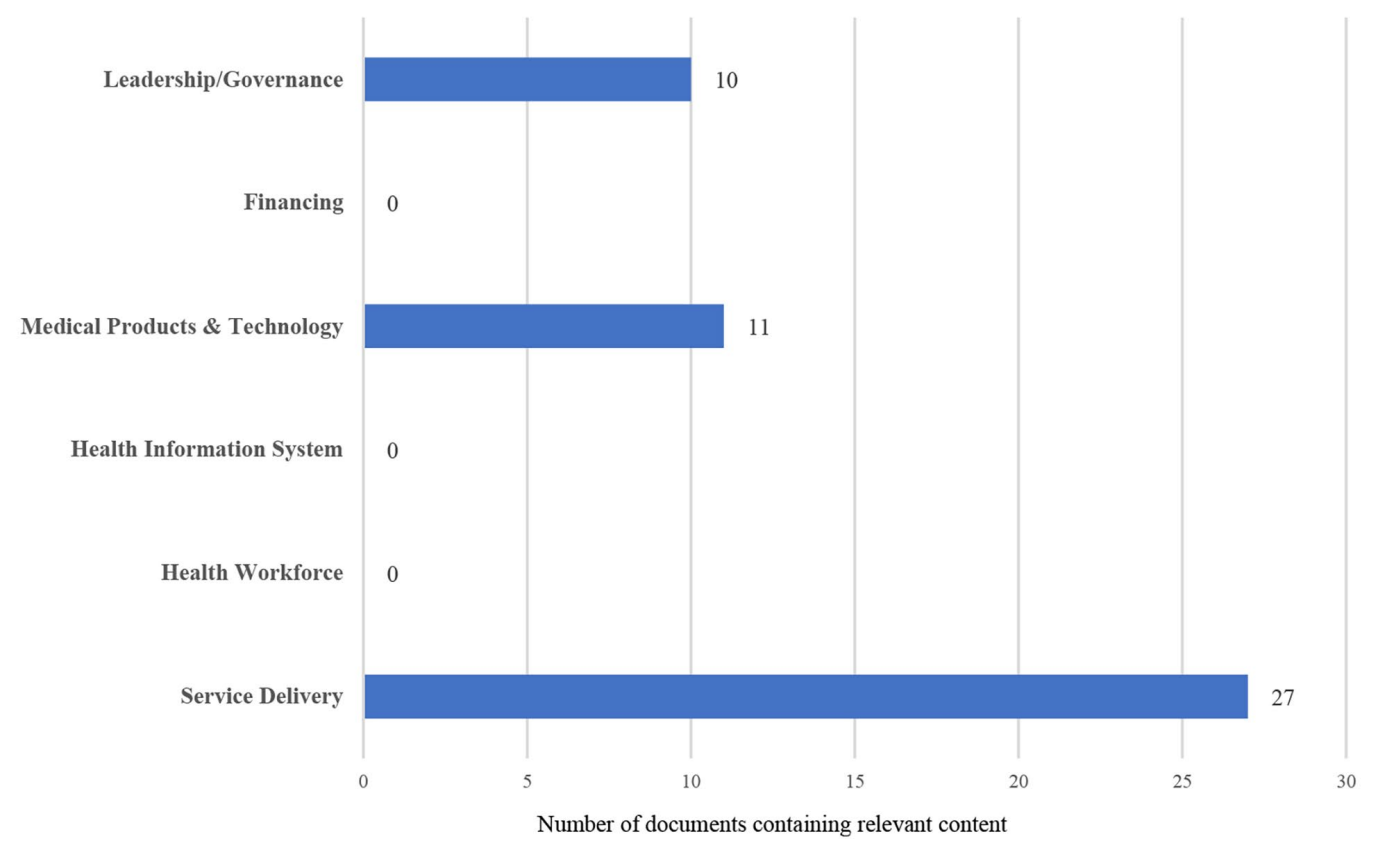
Summary of policy mapping results against the WHO Health System Building Blocks

Based on the summary of policy mapping results included in Fig. 3, the service delivery component of the health system was addressed in the content of most documents included. Essential access to medical products and technologies was reflected in the content of 11 documents. There were 10 documents covering content about the leadership and governance of the government. However, the components of the health workforce, information system and financing were barely embodied in any document included.

#### Content about leadership/governance

The included documents referring to the leadership and governance component mainly emphasised setting general goals of curbing the growth rate of obesity and overweight, ensuring the construction of a societal environment in favour of the prevention and control of obesity, and strengthening prevention and early intervention of obesity and overweight, especially among school-age children and adolescents. See the examples of relevant content extracted from the included documents in Table 3a below.

**Table 3a:** Major tools and strategies based on the WHO Health System Building Blocks identified in the document content: leadership/governance

Tools and Strategies Identified	Examples
• Setting general goals for curbing the growth rate of obesity and overweight	• Document No. 13 Outline of “Healthy China 2030”: “*The growth rate of overweight and obesity will slow down significantly*.” • Document No. 19 Opinions of the State Council on Implementing Healthy China Action: “The growth rate of adult obesity continues to slow through 2022 and 2030.” • Document No. 32 Implementation plan for prevention and control of obesity in children and adolescents (2020): “*Goal of prevention and control-national level: to take the average annual increase of overweight and obesity rates from 2002 to 2017 as the baseline, and reduce the average annual increase of overweight and obesity rates in children and adolescents aged 0–18 years by 70% from 2020 to 2030, laying the foundation for zero increase of overweight and obesity in children and adolescents*.”
• Ensuring the construction of a societal environment in favour of the prevention and control of obesity	• Document No. 32 Implementation plan for prevention and control of obesity in children and adolescents (2020): “*to construct a supportive environment for prevention and control of obesity to effectively curb the epidemic of overweight and obesity*.”
• Strengthening prevention and early intervention of obesity and overweight, especially among school-age children and adolescents	• Document No. 30 Basic Healthcare and Health Promotion Law: “*The state will incorporate health education into the national education system. Schools should make use of various forms of health education to popularise health knowledge, scientific fitness knowledge and first aid knowledge and skills, improve students’ awareness of active disease prevention, cultivate students’ good hygiene practices and healthy lifestyle, reduce and improve students’ myopia, obesity and other bad health conditions*.” • Document No. 35 Major Work Plans for Deepening the Reforms of the Medical and Health System of 2021: “*to promote the prevention and control of myopia and overweight and obesity among children and adolescents*”

#### Content about service delivery

At the service delivery level, general policy suggestions and proposals were put forward to provide and boost the screening and surveillance of overweight and obesity as a major risk factor for other non-communicable diseases (NCDs) as well as to enhance health education to raise the awareness of health consequences of obesity. Specific initiatives and programmes concerning childhood obesity were identified in this aspect, which mainly focused on dietary measures and physical activities. Table 3b contains concrete examples of content concerning each measure identified.

**Table 3b:** Major tools and strategies based on the WHO Health System Building Blocks identified in the document content: service delivery

Tools and Strategies Identified	Examples
• Providing and boosting the screening and surveillance of overweight and obesity as a major risk factor for other NCDs	• Document No. 6 China’s Chronic Disease Prevention and Control Work Plan (2012–2015): “*Sect. 4 Strategies and measures: to expand healthcare services, and to timely identify and manage high-risk groups. We will expand the coverage of basic public health services, and strengthen the detection and management of people at high risk of chronic diseases (high blood pressure, blood glucose and blood lipids, smoking, alcoholism, obesity and overweight)*.” • Document No. 14 “Thirteenth Five-Year” Medicine and Health Plan: “To s*trengthen prevention and control of major diseases - Implementing comprehensive prevention and control of chronic diseases: to comprehensively implement blood pressure measurement for people over 35 years old at first diagnosis, gradually carry out a risk assessment and intervention guidance for people at high risk of chronic diseases, such as elevated blood pressure and glucose, dyslipidemia, overweight and obesity, and to include oral health examination and pulmonary function test in routine physical examination*.” • Document No. 19 China’s Medium- and Long-Term Plan for the Prevention and Treatment of Chronic Diseases (2017–2025): “*Sect. 3 Strategies and Measures (2) to implement early diagnosis and intervention to reduce the risk of disease in the high-risk population. Personalised health interventions should be developed: community health service centres and township hospitals should gradually carry out a risk assessment and intervention guidance for people at high risk of chronic diseases, such as overweight and obesity, elevated blood pressure and blood glucose, and abnormal blood lipid, and provide counselling services such as balanced diet, physical activity, health care, and physique identification*.”
• Enhancing health education to raise the awareness of health consequences of obesity through specific diet-based or physical activity-oriented initiatives and programmes targeting children and adolescents	• Document No. 20 National Nutrition Plan (2017–2030): “*Major campaigns: Student nutrition Improvement action: Intervention for students’ overweight and obesity. Carry out “exercise + nutrition” weight management and intervention strategy for students, develop balanced diet and nutrition education for students, and enhance students’ physical exercise. Strengthen the management of food sales in and around the campus. Strengthen the monitoring and evaluation of overweight and obesity among students, analyse the influencing factors of family, school and society, and put forward targeted comprehensive intervention measures*.” • Document No. 33 Opinions on Stepping up Patriotic Health Campaigns and Efforts: “*A variety of education and teaching forms of health intervention should be provided for students to effectively prevent and control myopia, obesity, etc.*” • Document No. 40 Enhanced Action Plan for Healthy Children (2021–2025): “*to reduce sedentary time, promote balance between eating and activity, prevent and reduce overweight and obesity in children*.”

#### Content about medical products and technologies

In terms of medical products and technologies, promoting the innovation of therapeutic strategies and facilitating the extended application of appropriate medications and techniques were identified as the major efforts. In the past few years, significant progress in developing new anti-obesity drugs has been achieved. The drug approval authority in China also noticed this advancement and the activeness of domestic research. To facilitate innovation in the local market with huge unmet clinical needs, the NMPA issued two technical guiding principles on the clinical trials of anti-obesity drugs. In the 14th Five-Year (2021–2025), documents regarding the development of Traditional Chinese Medicine (TCM) proposed to demonstrate the notable strengths of TCM in the prevention and intervention of obesity by applying appropriate TCM procedures. See Table 3c for details.

**Table 3c:** Major tools and strategies based on the WHO Health System Building Blocks identified in the document content: essential access to medical products and technologies

Tools and Strategies Identified	Examples
• Encouraging the innovation of therapeutic strategies	• Document No. 44 Technical Guiding Principles for the Clinical Trials of Body Weight Control Drugs: “*In recent years, the research and development of weight control drugs have increased year by year in the world and China, becoming an active drug development field. However, there are no clinical trial guidelines for weight control drugs in China at present. To encourage and promote the research and development of weight control drugs, and standardise the clinical study design and related technical requirements, this guideline is formulated*.” • Document No. 40 Enhanced Action Plan for Healthy Children (2021–2025): “*Focusing on the key areas of prevention and control of childhood obesity and genetic and metabolic diseases, screening and intervention of children’s psychological and behavioural abnormalities, tertiary prevention of birth defects, comprehensive treatment of critical and severe children and comprehensive prevention and control of major diseases, we will vigorously develop children’s medical and healthcare technologies with independent intellectual property rights and in line with national conditions*.”
• Demonstrating the notable strengths of TCM in the prevention and intervention of obesity by applying appropriate TCM procedures • Facilitating the extended application of appropriate medications and techniques	• Document No. 50 Notice of State Council General Office on Printing and Distributing the “Fourteen Five-Year” Plan for the Traditional Chinese Medicine Development: “*To improve disease prevention capacity, efforts should be made to promote the health and to upgrade preventative treatment of diseases by using traditional Chinese medicine. The prevention and treatment of myopia, scoliosis, and obesity by applying appropriate TCM techniques in children and adolescents will be carried out*.” • Document No. 55 Implementation Plan for the Special Campaign on Promoting Healthy China: “*In response to health problems such as obesity and scoliosis among children and adolescents, pilot intervention programmes should be carried out by applying appropriate techniques of traditional Chinese medicine, health education activities on the prevention and control of obesity and scoliosis among children and adolescents with traditional Chinese medicine should be organised, so as to guide children and adolescents to develop a healthy individual lifestyle.*”

## Discussion

This documentary analysis comprehensively investigated national official documents and guidelines related to the prevention and control of obesity by the central government of China. Through a systematic search and screening procedure of relevant documents, we finally included 55 documents for analysis. It has been clear that in response to the rapid growth of overweight and obesity among its population in recent decades, the central government of China has introduced a growing number of legal, policy and administrative measures concentrating on three out of the WHO health system six building blocks, reflecting its firm resolve to promoting public health.

The thirteenth five-year plan period (2016–2020) was found to be a critical time node, as the figure of official documents and guidelines related to obesity showed a notable rise since then. Especially, the first year of this period witnessed the launching of the *Outline for the Healthy China 2030 Plan* and the “Thirteenth Five-Year” Medicine and Health Plan. They laid the cornerstones for prioritising health for all in all policies, integrating prevention and control of major diseases including NCDs, and emphasising the significance of a healthy lifestyle in the awareness-raising health promotion. In accordance, specific strategies, implementation plans, and large-scale health promotion campaigns were rolled out consecutively to address the prevention and control of NCDs and obesity. For instance, *China’s Medium- and Long-Term Plan for the Prevention and Treatment of Chronic Diseases (2017–2025)* emphasised the importance of early diagnosis and intervention to reduce the risk of disease in the high-risk population, which includes those with overweight and obesity. *Implementation Plan for Prevention and Control of Obesity in Children and Adolescents (2020)* requires governments at all levels to give full play of their roles in constructing a supportive environment for the prevention and control of obesity in children and adolescents. These policy actions at the top design and implementation levels in good coherence implied the determination of the China government to enable healthy living for all.

In terms of key policymakers, the State Council and its affiliated ministries played a pivotal role in releasing the official documents. In particular, the National Health Commission was the dominant operator in aligning resources and different government sectors and stakeholders for formulating relevant policy documents and guidelines. Additionally, a clear cross-departmental policy-making process was observed in eight of the documents.

The results from the content analysis through the lens of the WHO Health System Building Blocks framework demonstrated that there is great room for the government to push the health system further forward from all six aspects to overcome the prevalent challenge.

Based on our analysis, the government has devoted greater attention to preventing and controlling obesity by leveraging various policy approaches in the past few years. Currently, the government set goals of attenuating further growth of obesity and overweight in the general population and strengthening the prevention and early intervention of obesity and overweight, especially among school-age children and adolescents. Moreover, it released general implementation plans to improve the obesogenic environment. The government may further strengthen its pivotal role in the multi-sectoral coordination in policy making and implementation. In our review of the existing documents, we could see the emergence of the cross-department pattern. However, there is a lack of specific policies or integrated policy systems centring around obesity with great convergence and coherence to mobilise all the stakeholders to make a fundamental change.

In its report on the project of *Ending Childhood Obesity Implementation Plan*, WHO stressed the “ultimate responsibility” of the government for securing healthy life for its whole population, which requires sustainable high-level engagement and leadership [46]. In the countries that have been confronted with the obesity epidemic for a longer period, a system approach embracing multi-sectoral efforts is the cross-cutting theme in the national policies to reverse the obesity trend by enabling the population to access healthy behaviours [39, 47–49]. A flagship national planning was the Foresight Obesities initiated during the Blair administration to provide an evidence-informed change-making opportunity for the government in the next 40 years [37]. One of the key outcomes of this forward-thinking government project was to make a structural change in the narrative of obesity from a simplified blame of individual self-control to a complexity of physiological and socio-economic-environmental factors. It also drew an analogy between tackling obesity and climate change to highlight the necessity of taking a whole-system approach to incorporate the efforts of governments, industries, communities, and individuals. With the system modelling technique, an iconic systems map was generated to visualise the multi-faceted determinants of the ongoing obesity epidemic in the UK. This qualitative obesity called for collective responsibilities and cross-sectoral collaboration of various government departments. However, in the practice and follow-up implementations, this framing of obesity did not prevail over the conventional one with the locus of the individual even before the project was phased out. More recently, Australia launched the *National Obesity Strategy Plan 2022–2032* to strengthen and reorient the whole system to prevent the further increase of obesity prevalence in the country with the ambition to “create supportive, sustainable and healthy environments” at the macro and meso levels, “empower people to stay healthy” at the micro level, and “ensure access to early intervention and care” as an essential downstream approach [50]. The effective implementation of the plan was intended to be realised through strong leadership of the government, an evidence-based decision-making process, and enduring investment in the existing fiscal system through innovative funding mechanisms that can proportionally reflect the burden of obesity onto society. The evaluation of accountability of the strategic plan was also embedded in the monitoring mechanism. These two examples with strengths and gaps may provide some meaningful reference for the future strengthening of the health system against obesity. The government should devote a continuous and greater commitment and input in the top design to adopt a sustainable system approach to tackling the obesity epidemic.

To enhance the effectiveness and quality of health service delivery, the government made efforts in promoting the screening and surveillance of overweight and obesity as a major risk factor for other NCDs and introducing specific preventative initiatives and programmes with a clear orientation towards optimising nutrition intake and energy consumption via physical activities and a strong favour of children and adolescents. The government enables essential access to medical products and technologies to combat obesity through two major tracks. One is to facilitate access to novel pharmaceutical options with solid evidence of clinical effectiveness and safety on the China market by releasing technical guiding principles on relevant clinical trials with an indication of obesity. The other is to encourage to play the strength of TCM in the prevention and management of chronic diseases by applying appropriate TCM and techniques to treat obesity.

Among the established therapeutic options for obesity, lifestyle modification is recommended as the single first-line choice [51–53]. Pharmacological therapy could be conditionally applied to patients who could not achieve meaningful weight loss after a prescribed period of guided lifestyle intervention. Surgical procedures have progressed to reach an effective reduction of body weight with proven safety, but only for patients with severe or morbid obesity. Regarding the anti-obesity drugs, there is only one AOD for long-term use approved on the China market, and five on the American market in the past two decades [54, 55].

Exceptional advancements are seen in the recent development of novel anti-obesity drugs [56–58]. In particular, the GLP-1-based therapeutic strategies stood out with record-setting weight loss effects and relatively desirable safety performance so far in clinical trials at different phases [59, 60]. The Chinese biopharmaceuticals have also played catch-up in this arena. There are at least 13 clinical trials of various new-generation AODs at different phases that are undertaken in China. This scenario has prompted the regulatory authorities to change their risk-benefit calculations, as in history several candidates failed the approval due to unexpected severe adverse effects. Liraglutide is the first GLP-1-based medicine approved for obesity in the US. In 2021, Semaglutide was approved for the indication of weight loss in both the American and European markets. In the same year, the China NMPA issued two technical guiding principles consecutively on AODs. The potential shift in the accessibility of novel AODs with outstanding and safe weight loss effects for chronic use may eventually bring certain changes in the relevant policies and guidelines on the prevention and management of obesity.

In the meanwhile, the *Outline of the Fourteenth Five-Year Plan* initiated special support to be provided to traditional Chinese Medicine, which has great and unique strength in the prevention and treatment of chronic diseases. Concerning obesity, both of the *“Fourteen Five-Year” Plan for the Traditional Chinese Medicine Development* and the *Implementation Plan for the Special Campaign on Promoting Healthy China* promoted the application of appropriate TCM in the prevention and treatment of childhood and adolescent obesity. It is worth systematically evaluating the clinical evidence on the effectiveness of different TCM-based techniques (e.g., acupuncture, medication, and physical to inform practice and policymaking to play the unique strength of TCM.

Our analysis revealed that the current response is still inadequate, as there is a lack of explicit measures within the current system to combat obesity from the aspects of health financing, information system and health workforce. To strengthen the integrity of the health system to reverse the obesity trend in China, future efforts should be made in the following aspects. First, health financing tools could be mobilised, which would mainly relate to the health insurance coverage of healthcare services provided to patients with obesity and continuous fiscal investment in the large-scale preventative programmes and healthcare services; b. to make better use of the available surveillance mechanism for monitoring the NCDs to closely monitor the obesity prevalence and incidence in a larger population, which could vigorously inform the follow-up implementation of timely prevention and early intervention measures; and c. to empower health professionals to provide evidence-informed health advice and clinical practice to patients through training and upskilling, especially to raise their recognition of the complexity and health consequences of obesity.

There are two potential major limitations of this study that are worth noting. First, this study mainly placed focus on the national-level policy actions to tackle the obesity issue. In China, more specific policies, implementation plans, and initiatives are often formulated and carried out at the provincial level. Exclusion of this part of policy responses in different provinces may hinder us from obtaining a complete picture of the management of obesity. Second, this analysis based on policy documents may only constitute an exploratory effort to understand China’s readiness and responses to the challenge of obesity. More extensive investigation in a multi-method approach could be conducted in conjunction to seek solid triangulation of evidence from diversified sources for future policy implications [61].

## Conclusions

The unprecedented obesity prevalence has triggered China’s health system to put a growing emphasis on its prevention and management. Our analysis revealed that the current endeavours concentrated on the affirmation of the government’s role in combating the disease, improving healthcare service delivery, and promoting access to medical products and technologies. In the future, the government should not only continue to intensify its efforts from the above three aspects, but also seek to advance the development in health financing, information system, and workforce to strengthen its health system with high integrity to overcome the obesity epidemic.

## Electronic supplementary material

Below is the link to the electronic supplementary material.

Supplementary File 1: List of Affiliated Ministries of the State Council of the People’s Republic of China

Supplementary File 2: National Documents About Obesity Prevention and Control in China

## Data Availability

The datasets used and/or analysed during the current study available from the corresponding author on reasonable request.

## List of Abbreviations

HPSR: Health policy and systems research
WHO: World Health Organization
NPC: National People’s Congress
TCM: Traditional Chinese medicine
NMPA: National Medical Products Administration
AOD: Anti-obesity drugs
