# Supplementary material for: Policy analysis of system responses to addressing and reversing the obesity trend in China: a documentary research
Source: BMC Public Health. 2023 Jun 21;23:1198. doi: 10.1186/s12889-023-15890-7 (PMC10283163; doi:10.1186/s12889-023-15890-7)
Supplement: Supplementary file 1 — List of Affiliated Ministries of the State Council of the People’s Republic of China [file 12889_2023_15890_MOESM1_ESM.docx]

**Supplementary File 1: *List of Affiliated Ministries of the State Council of the People’s Republic of China***

| Ministry of Foreign Affairs | Ministry of Public Security | Ministry of Ecology and Environment |
| --- | --- | --- |
| Ministry of National Defense | Ministry of State Security | Ministry of Housing and Urban-Rural Development |
| National Development and Reform Commission | Ministry of Civil Affairs | Ministry of Transport |
| Ministry of Education | Ministry of Justice | Ministry of Water Resources |
| Ministry of Science and Technology | Ministry of Finance | Ministry of Agriculture and Rural Affairs |
| Ministry of Industry and Information Technology | Ministry of Human Resources and Social Security | Ministry of Commerce |
| National Ethnic Affairs Commission | Ministry of National Resources | Ministry of Culture and Tourism |
| National Health Commission | Ministry of Veteran Affairs | Ministry of Emergency Management |
| People’s Bank of China | National Audit Office |  |
