# Supplementary material for: Policy analysis of system responses to addressing and reversing the obesity trend in China: a documentary research
Source: BMC Public Health. 2023 Jun 21;23:1198. doi: 10.1186/s12889-023-15890-7 (PMC10283163; doi:10.1186/s12889-023-15890-7)
Supplement: Supplementary file 2 — National Documents About Obesity Prevention and Control in China [file 12889_2023_15890_MOESM2_ESM.docx]

**Supplementary File 2: National Documents About Obesity Prevention and Control in China**

| **No** | **Document Title in English** | **Document Title in Chinese** | **Year of Issue** |
| --- | --- | --- | --- |
| **Prior to the 12^th^ Five-Year (before 2011)** | |  |  |
| **1** | Guidelines for Prevention and Control of Overweight and Obesity in the Chinese Adults (2003) | 中国成人超重和肥胖症防治指南（2003） | 2003 |
| **2** | Guidelines for Prevention and Control of Overweight and Obesity in Chinese School-age Children and Adolescents (Trial) | 中国学龄儿童少年超重和肥胖预防与控制指南（试用）2007年 | 2007 |
| **3** | Management Measures for Nutrition Improvement | 营养改善工作管理办法 | 2010 |
| **The 12^th^ Five-Year Plan Period (2011-2015)** | |  |  |
| **4** | Outline of Chinese Child Development (2011-2020) | 中国儿童发展纲要（2011-2020年） | 2011 |
| **5** | National Fitness Program (2011-2015) | 全民健身计划（2011-2015年） | 2011 |
| **6** | China's Chronic Disease Prevention and Control Work Plan (2012-2015) | 中国慢性病防治工作规划（2012-2015年） | 2012 |
| **7** | Technical Specifications for the Management of Nutritional Diseases in Children" (including "Technical Specifications for the Guidance of Child Feeding and Nutrition" | 儿童营养性疾病管理技术规范（含《儿童喂养与营养指导技术规范） | 2012 |
| **8** | Maternal and Child Health Literacy-Basic Knowledge and Skills (Trial) | 母婴健康素养--基本知识与技能（试行） | 2012 |
| **9** | Outline of Food and Nutrition Development in China (2014-2020) | 中国食物与营养发展纲要（2014-2020年） | 2014 |
| **10** | National Student Physical Health Standard (Revised in 201 | 国家学生体质健康标准（2014年修订） | 2014 |
| **11** | Chinese Citizens' Health Literacy, Basic knowledge and Skills Definitions (2015) | 中国公民健康素养--基本知识与技能（2015年版） | 2015 |
| **12** | Report on Chinese Residents’ Chronic Diseases and Nutrition 2015 | 《中国居民营养与慢性病状况报告（2015年）》 | 2015 |
| **The 13^th^ Five-Year Plan Period (2016-2020)** | |  |  |
| **13** | Outline of Healthy China 2030" Plan | “健康中国2030”规划纲要 | 2016 |
| **14** | "Thirteenth Five-Year" Medicine and Health Plan | “十三五”卫生与健康规划 | 2016 |
| **15** | Medium- and Long-Term Plan for the Youth Development (2016-2025) | 中长期青年发展规划（2016-2025年） | 2016 |
| **16** | Guidance on strengthening health promotion and education | 关于加强健康促进与教育的指导意见 | 2016 |
| **17** | Dietary Guidelines for Chinese Residents (2016) | 中国居民膳食指南（2016年） | 2016 |
| **18** | Dietary Guidelines for Pregnant Women (2016) | 孕期妇女膳食指南（2016年） | 2016 |
| **19** | China's Medium- and Long-Term Plan for the Prevention and Treatment of Chronic Diseases (2017-2025) | 中国防治慢性病中长期规划（2017-2025年） | 2017 |
| **20** | National Nutrition Plan (2017-2030) | 国民营养计划（2017-2030） | 2017 |
| **21** | Health Action Plan for All (2017-2025) | 全民健康生活方式行动方案（2017-2025年） | 2017 |
| **22** | Guidelines for National Physical Fitness | 全民健身指南 | 2017 |
| **23** | Action Plan for Healthy Children (2018-2020) | 健康儿童行动计划（2018-2020年） | 2018 |
| **24** | Core Information and Interpretation of Chinese Youth Health Education | 中国青少年健康教育核心信息及释义（2018版） | 2018 |
| **25** | Screening for Overweight and Obesity in School-age Children (WS / T 586-2018) | 卫生行业标准--学龄儿童青少年超重肥胖筛查（WS/T 586-2018） | 2018 |
| **26** | Guidelines for Consumption of Snacks for Chinese Children and Adolescents | 中国儿童青少年零食消费指南 | 2018 |
| **27** | School Food Safety and Nutrition and Health Management Regulations | 学校食品安全与营养健康管理规定 | 2019 |
| **28** | Healthy China Initiative (2019-2030) | 健康中国行动（2019-2030年） | 2019 |
| **29** | Opinions of the State Council on Implementing Healthy China Action | 关于实施**健康中国行动**的意见 | 2019 |
| **30** | Basic Healthcare and Health Promotion Law | 中华人民共和国基本医疗卫生与健康促进法 | 2019 |
| **31** | Notice of State Council General Office on Printing and Distributing the Outline for the Construction of a Powerful Country in Sports | 国务院办公厅关于印发体育强国建设纲要的通知 | 2019 |
| **The 14^th^ Five-Year Plan Period （2021-2025）** | |  |  |
| **32** | Implementation plan for prevention and control of obesity in children and adolescents (2020) | 儿童青少年肥胖防控实施方案（2020） | 2020 |
| **33** | Opinions on Stepping up Patriotic Health Campaigns and Efforts | 关于深入开展爱国卫生运动的意见 | 2020 |
| **34** | Report on Chinese Residents’ Chronic Diseases and Nutrition 2020 | 《中国居民营养与慢性病状况报告（2020年）》 | 2020 |
| **35** | Major Work Plans for Deepening the Reforms of the Medical and Health System of 2021 | 深化医药卫生体制改革2021年重点工作任务 | 2021 |
| **36** | "Fourteenth Five-Year" Plan for Establishing the National Capacity of Clinical Specialties | “十四五”国家临床专科能力建设规划 | 2021 |
| **37** | Special actions such as "three reductions and three health benefits" | 持续推进“三减三健”专项行动重点工作 | 2021 |
| **38** | Implementation Plan for Consolidating and Building on the Effective Connection Between the Achievements of Poverty Alleviating Efforts Through Healthcare and Rural Revitalisation | 巩固拓展健康扶贫成果同乡村振兴有效衔接实施意见 | 2021 |
| **39** | Notice of the NHC on Holding Series of Awareness Days of Non-Communicable Diseases | 国家卫生健康委疾控局关于开展2021年慢性病系列宣传日活动的通知 | 2021 |
| **40** | Enhanced Action Plan for Healthy Children (2021-2025) | 健康儿童行动提升计划（2021-2025年） | 2021 |
| **41** | Outline of Chinese Children Development (2021-2030) | 中国儿童发展纲要（2021-2030年） | 2021 |
| **42** | Outline of Chinese Women Development (2021-2030) | 中国妇女发展纲要（2021） | 2021 |
| **43** | National Fitness Program (2016 ~ 2020) | 全民健身计划（2021-2025年） | 2021 |
| **44** | Technical Guiding Principles for the Clinical Trials of Body Weight Control Drugs | 体重控制药物临床试验技术指导原则 | 2021 |
| **45** | Management Measures of Health Examination in Primary and Secondary Schools (2021） | 中小学健康体检管理办法（2021年版） | 2021 |
| **46** | China Blue Paper on Obesity Prevention and Control | 中国肥胖预防和控制蓝皮书 | 2021 |
| **47** | Expert Consensus on the Procedure of Body Weight Management Among Patients with Overweight or Obesity | 超重或肥胖人群体重管理规范专家共识（2021） | 2021 |
| **48** | Guidelines for Prevention and Control of Overweight and Obesity in Chinese Children (Trial) | 儿童肥胖预防与控制指南（2021） | 2021 |
| **49** | Guidelines for Prevention and Control of Overweight and Obesity in the Chinese Adults (2021) | 中国成人超重和肥胖预防控制指南（2021） | 2021 |
| **50** | Notice of State Council General Office on Printing and Distributing the "Fourteen Five-Year" Plan for the Traditional Chinese Medicine Development | 国务院办公厅关于印发“十四五”中医药发展规划的通知 | 2022 |
| **51** | "Fourteenth Five-Year" National Health Plan | “十四五”国民健康规划 | 2022 |
| **52** | "Fourteenth Five-Year" Plan for Promoting the Health Standardisation Work | “十四五”卫生健康标准化工作规划 | 2022 |
| **53** | Guiding Principles for the Design of Clinical Trials of Liraglutide for Weight Management | 利拉鲁肽用于体重管理的临床试验设计指导原则 | 2022 |
| **54** | Dietary Guidelines for Chinese Residents (2022) | 中国居民膳食指南（2022年） | 2022 |
| **55** | Implementation Plan for the Special Campaign on Promoting Healthy China | 健康中国行动中医药健康促进专项活动实施方案 (2022） | 2022 |
